# Identifying changes in immune cells and constructing prognostic models using immune-related genes in post-burn immunosuppression

**DOI:** 10.7717/peerj.12680

**Published:** 2022-01-13

**Authors:** Peng Wang, Zexin Zhang, Bin Yin, Jiayuan Li, Cheng Xialin, Wenqin Lian, Yingjun Su, Chiyu Jia

**Affiliations:** 1 Department of Burns and Plastic & Wound Repair Surgery, Xiang’an Hospital of Xiamen University, School of Medicine, Xiamen University, Xiamen, China; 2 Department of Anesthesia Operation, Xiang’an Hospital of Xiamen University, School of Medicine, Xiamen University, Xiamen, Fujian, China; 3 Department of Burns and Plastic Surgery, Plastic Surgery Hospital, Xi’an International Medical Center, Xi’an, Shaanxi, China

**Keywords:** Post-burn immunosuppression, Prognostic biomarkers, Targets of immunotherapy, Immune-related cell, miRNA

## Abstract

**Background:**

Burn patients are prone to infection as well as immunosuppression, which is a significant cause of death. Currently, there is a lack of prognostic biomarkers for immunosuppression in burn patients. This study was conducted to identify immune-related genes that are prognosis biomarkers in post-burn immunosuppression and potential targets for immunotherapy.

**Methods:**

We downloaded the gene expression profiles and clinical data of 213 burn patients and 79 healthy samples from the Gene Expression Omnibus (GEO) database. Immune infiltration analysis was used to identify the proportion of circulating immune cells. Functional enrichment analyses were carried out to identify immune-related genes that were used to build miRNA-mRNA networks to screen key genes. Next, we carried out correlation analysis between immune cells and key genes that were then used to construct logistic regression models in GSE77791 and were validated in GSE19743. Finally, we determined the expression of key genes in burn patients using quantitative reverse transcription polymerase chain reaction (qRT-PCR).

**Results:**

A total of 745 differently expressed genes were screened out: 299 were up-regulated and 446 were down-regulated. The number of Th-cells (CD4+) decreased while neutrophils increased in burn patients. The enrichment analysis showed that down-regulated genes were enriched in the T-cell activation pathway, while up-regulated genes were enriched in neutrophil activation response in burn patients. We screened out key genes (*NFATC2, RORA*, and *CAMK4*) that could be regulated by miRNA. The expression of key genes was related to the proportion of Th-cells (CD4+) and survival, and was an excellent predictor of prognosis in burns with an area under the curve (AUC) value of 0.945. Finally, we determined that *NFATC2, RORA*, and *CAMK4* were down-regulated in burn patients.

**Conclusion:**

We found that *NFATC2, RORA*, and *CAMK4* were likely prognostic biomarkers in post-burn immunosuppression and potential immunotherapeutic targets to convert Th-cell dysfunction.

## Introduction

According to the World Health Organization (WHO), an estimated 11 million burns and 180,000 resulting deaths occur worldwide each year. Although some attempts have been made to use white blood cells, platelets, cytokines, burn areas, score scales, and other methods to evaluate the prognosis of these incidents, the accuracy is unsatisfactory and the application has been subjective (Osuka et al., 2019; Kraft et al., 2012; Prasad, Thode & Singer, 2020; Hur et al., 2015). Although changes at the gene level are subtle and precise and have been used to predict the prognosis of many diseases (Chang et al., 2020; Li et al., 2020), few studies have explored the exact relationship between immune-related genes (IRGs) and burn deaths. Furthermore, previous research has shown that >60% of deaths are related to infection-related complications in burn patients, mainly because of antibiotic resistance (Jeschke et al., 2020; Burns, 2020; Mofazzal Jahromi et al., 2018; Vinaik et al., 2019; Lachiewicz et al., 2017). Immune dysfunction after burn injury is an important cause of infection. Immunotherapy that activates T-cells by targeting T-cell factors can effectively improve immune function (O’Sullivan et al., 1995; Kinoshita et al., 2013; Kinoshita et al., 2011; Toliver-Kinsky et al., 2003). However, relatively few studies have researched how to identify the key molecules of systematic immunosuppression after burns. Therefore, identifying RNA changes in post-burn patients is important when exploring prognostic biomarkers and potential immunotherapy targets.

*In vivo*, proteins are translated from mRNA that can be regulated by miRNAs through exosomes that are secreted by stem cells (Hu et al., 2019; Mori et al., 2019). After burn injury, exosomes containing miR-181c that is secreted from mesenchymal stem cells can ameliorate the excessive inflammatory response by suppressing the TLR4 signaling pathway through the down-regulation of NF-κB and p-p65 protein expression (Li et al., 2016). Additionally, miRNAs can regulate the pathophysiological process of infection in burn patients by destroying or protecting the intestinal mucosal barrier. MiR-320 prevents the apoptosis of intestinal cells by down-regulating the target gene, PTEN (Ke et al., 2019). Conversely, miR-150 can regulate IL-6 and KC protein expression levels and cause damage to the intestinal mucosal barrier after burns, leading to mucosal immune destruction and thereby inducing infection (Morris et al., 2017). Previous studies suggest that mRNAs could be regulated by miRNAs to regulate systemic immune homeostasis and modulate anti-infective effects after burns through exosomes secreted by stem cells. IRGs have a clear association with infection, which is the leading cause of burn-related death. Furthermore, the interaction between mRNA and miRNA has proved to play a vital role in a variety of diseases and has reliable prognostic predictive ability (Chang et al., 2020; Li et al., 2020). However, to our knowledge, no study has yet revealed a relationship between immune-related genes and prognosis. Other studies have identified the differential expression of miRNAs (DEMs) in thermal-stimulated epidermal stem cells, but did not determine the target gene of miRNAs or whether those RNA were associated with prognosis.

**Figure 1:**
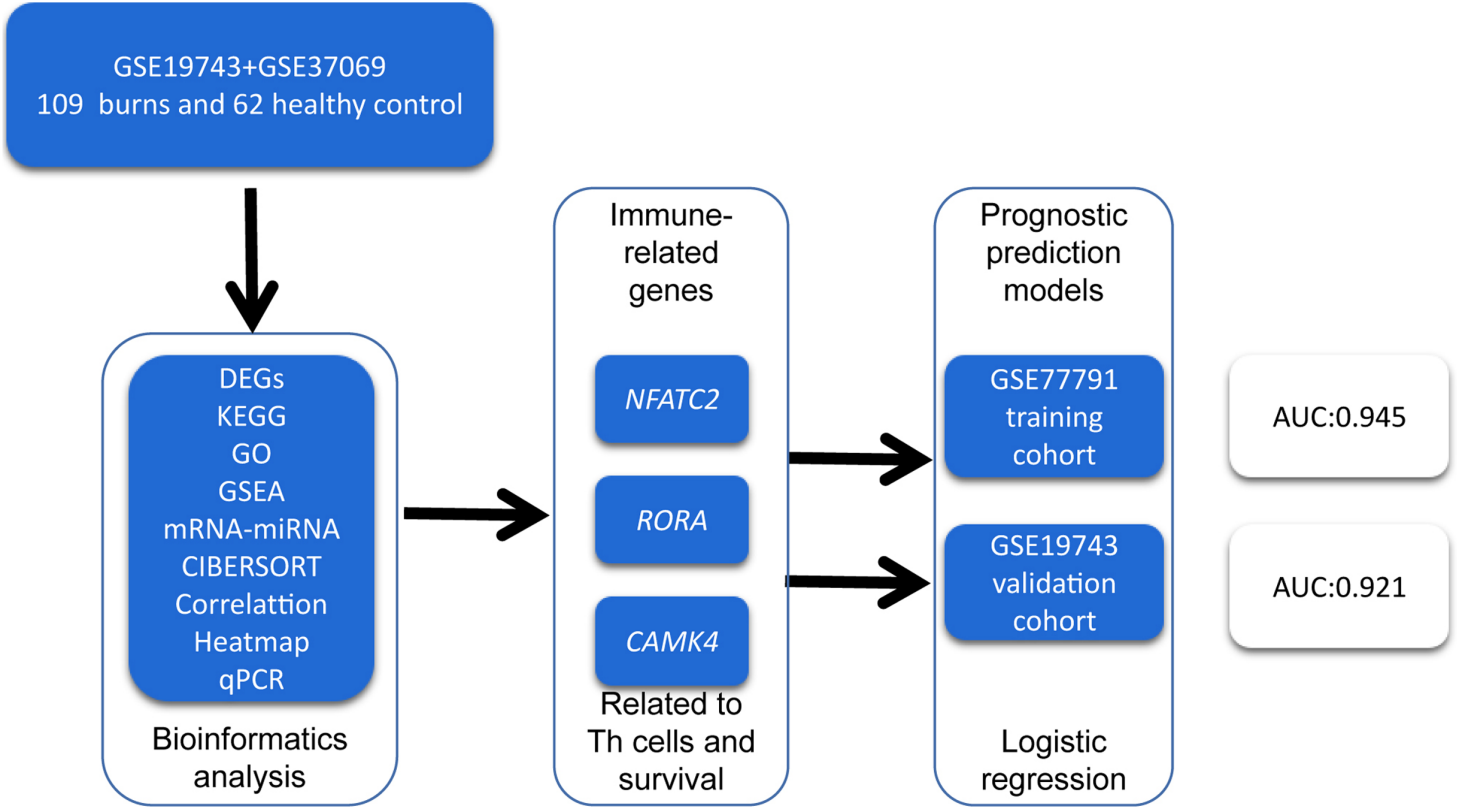
A graphical summary of the research design.

In order to identify prognosis biomarkers and potential immunotherapy RNAs, we first screened the differentially expressed genes (DEGs) in the RNA sequence of blood samples from 213 burn patients and 79 healthy adults using bioinformatics methods. We investigated the changes in circulating immune cells (CICs) and the functions of DEGs in burn patients. We then identified key genes related to the proportion and prognosis of CICs that may be potential biomarkers for immunotherapy and prognosis in post-burn immunosuppression. A graphical summary is presented in Fig. 1.

## Methods

### Acquisition of RNA data

We downloaded two microarray expression profiles of human whole blood samples from burn patients GSE19743 and GSE37069 from the Gene Expression Omnibus (GEO) database (http://www.ncbi.nlm.nih.gov/geo/). Patients >18 years or <55 years with a sampling time between 280 and 705 h were included (Table 1). We selected 28 and 81 burn patients with control groups of 25 and 37 healthy controls for the GSE19743 and GSE37069 data sets (screening DEGs), respectively. Next, we used the expression profiles and clinical data (survival and non-survival) from the GSE19743 and GSE77791 gene sets (training and validating prognostic models), with 161 blood samples that included 81 survival and 23 non-survival (clinical data are shown in Table 2). We also selected data on the DEMs in epidermal stem cells after thermal stimulation from a previously published article (Rong & Liu, 2020) in PubMed. Given that all data were available from public databases, there was no need for patient consent or ethical approval.

**Table 1:** Clinical data from burn patients in GSE37069 and GSE19743 (datasets utilized to screen differentially expressed genes).

Group (burn)	Sex			Age	Time of sampling
	*N*	Male	Female		
GSE19743	28	24	4	37.61 ± 7.98	439.28 ± 117.86
GSE37069	81	57	24	37.41 ± 10.45	411.92 ± 124.76
*p*	0.109			0.271	0.311

**Table 2:** Clinical data from burn patients in GSE37069 and GSE19743 (datasets utilized to screen differentially expressed genes).

Group	Sex			Age	TBSA		Time of sampling (GSE19743)
	*N*	Male	Female		Severe (30–49)	Major (49–100)	
Death	23	19	4	40.73 ± 7.67	3	20	422.24 ± 122.32
Survival	81	70	11	40.67 ± 10.74	21	60	393.33 ± 113.19
*p*	0.147			0.974	0.001		0.544

### Data processing

Using the R software package “Affy”, we performed the background correction of expression value and normalization of expression profile data, as well as conversion of the original data format, supplementation of missing values, background correction, and data standardization by the quantile method. We used “LIMMA” package in R software (version 4.0.5) to analyze common DEGs in the GSE19743 and GSE37069 data sets (FDR < 0.05, |logFC| > 1.5) and divided the common DEGs into two groups: the common up DEGs and the common down DEGs.

### Immune infiltration analysis

“CIBERSORT” is a machine learning algorithm that can analyze the proportion of immune cells using RNA-seq (Newman et al., 2015). We downloaded the GSE19743 expression profile, selected 28 burn patients and 25 healthy controls, and performed immune infiltration analysis using the “CIBERSORT” package in R software. We considered cells with *p* < 0.05 as CICs for further analysis. Next, we used the “ggplot2” package in R software to visualize the different proportion of CICs between burn patients and healthy controls.

### Enrichment analysis

We used the DAVID 6.8 online tool (https://david.ncifcrf.gov) to perform enrichment analysis with the Kyoto Encyclopedia of Genes and Genomes (KEGG) and Gene Ontology (GO) on the two groups of DEGs. The “ggplot2” package in R software was used to draw a bubble chart. ClueGo is a Cytoscape plug-in that can be used to perform enrichment analysis and visualize the results. We uploaded the common up DEGs as Cluster 1 and the common down DEGs as Cluster 2, and we performed biology process enrichment analysis in ClueGO. Finally, we constructed the interaction network between the enrichment results (FDR < 0.05).

### Construction of immune-related gene sets

Gene set enrichment analysis (GSEA) was utilized to detect whether the preset gene sets were enriched at the top or bottom of the sequencing table in order to identify the function of DEGs. We selected the microarray expression profiles of 28 burn patients and 25 healthy adults in the GSE19743 gene set and used the “GeoTcgaData” package in R software to convert the names of the gene probes to construct a gene expression matrix. The matrix was uploaded to the GSEA software for enrichment analysis. Finally, we selected immune-related genes (IRGs) in the biology process enrichment analysis in GO, KEGG, and GSEA to construct an IRG set for subsequent analysis.

### Constructing an miRNA–mRNA network and common expression analysis between key genes and key CICs

We obtained DEMs from epidermal stem cells under thermal stimulation referencing Rong & Liu (2020) and using the mirwalk online prediction tool (http://mirwalk.umm.uni-heidelberg.de/) to predict target miRNA genes. We simultaneously selected the target genes predicted by the three databases (miRWalk, Targetscan, and miRDB), among which the interactions between miRNA and mRNA in the Targetscan database were verified by experiments, for further research. We took the intersection of the predicted miRNA target genes and IRGs and performed correlation analysis between the genes and CICs to select key genes.

### Constructing a survival-related risk regression model and validating across different gene sets

We compared the differences in the expression profiles of key genes between the survival group and the nonsurival group in order to explore the correlation between the key genes and survival. SPSS software was utilized to draw the receiver operating characteristic (ROC) curve between the expression of key genes and survival status, and we calculated the AUC value to investigate the prognostic ability of key genes. Next, we validated the expression level of key genes and repeated the ROC curve results in the GSE77791 dataset. Then, we selected related genes to construct a logistic regression model and draw a nomogram using the training cohort GSE77791 (the logistic regression results are shown in Tables 3 and 4). Calibration curves and ROC curves were carried out to estimate the accuracy and discrimination of the nomogram. Finally, we validated the model using the external validation cohort GSE19743.

**Table 3:** Univariate logistic regression analysis.

Factors	*β*	*p*	OR	95% CI
NFATC2	−5.161	1.235E−07	0.006	[0.001–0.039]
RORA	−0.851	7.000E−06	0.427	[0.295–0.618]
CAMK4	−5.268	6.053E−09	0.004	[0.001–0.024]
CAMK2D	−1.278	9.600E−05	0.276	[0.145–0.527]
TBSA (50%)	1.483	0.008	4.407	[1.466–13.254]
Sex (1 = Female)	0.434	0.278	0.648	[0.296–1.419]
Age	0.011	0.270	1.011	[0.991–1.032]

**Table 4:** Multivariate logistic regression analysis.

Factors	*β*	*p*	OR	95% CI
NFATC2	−4.714	4.100E−05	0.006	[0.001–0.085]
RORA	−0.896	4.000E−03	0.427	[0.223–0.748]
CAMK4	−2.799	8.000E−03	0.004	[0.008–0.488]
CAMK2D	0.077	0.781	–	–
TBSA (>50%)	1.332	0.248	–	–

### Quantitative reverse transcription polymerase chain reaction (qRT-PCR)

Blood samples were collected from six burn cases and six healthy controls from the Department of Burns and Plastic & Wound Repair Surgery, Xiang’an Hospital of Xiamen University between December 1, 2019 and December 1, 2020. This experiment was conducted in accordance with the Declaration of Helsinki and with the approval of the Medical Ethics Committee of Xiang’an Hospital Affiliated with Xiamen University (xmuxayyky-2019-121) and informed patient consent in writing. Peripheral blood mononuclear cells (PBMC) were isolated from blood using Ficoll sodium diatrizoate gradient centrifugation (Sigma-Aldrich, St. Louis, MO, USA) and were dissolved in TRIzol reagent (Invitrogen, Carlsbad, CA, USA). The total RNA was extracted using a RNeasy kit (Qiagen, Hilden, Germany) and stored at −80 °C. Quantitative real time-PCR (qRT-PCR) was used to determine the quantitative expression of key genes in the burn patients. Reverse transcription was conducted using a 1st Strand cDNA Synthesis Kit (Takara Kusatsu, Shiga, Japan) at 42 °C for 60 min, followed by 95 °C for 5 min. A LightCycler® 480 II real-time PCR system (Roche, Basel, Switzerland) with a SYBR® Premix Ex Taq™ Kit (Takara) was used to perform PCR at 95 °C for 2 min, followed by 38 cycles at 95 °C for 30 s, 53 °C for 30 s, and 72 °C for 30 s. The results were normalized to U6. The relative number of genes normalized to control was calculated using the equation 2^–ΔΔCT^. The primer sequences are shown in Table 5.

**Table 5:** List of primers used in reverse transcription-quantitative PCR.

Gene name	Real-time quantitative PCR primer (5′to 3′)
*NFATC2*	F: 5′-CGATTCGGAGAGCCGGATAG-3′
	R: 5′-TGGGACGGAGTGATCTCGAT-3′
*RORA*	F: 5′-AAAAACATGGAGTCAGCTCCG-3′
	R: 5′-AGTGTTGGCAGCGGTTTCTA-3′
*CAMK4*	F: 5′-ACA GAT GCA AAC AGA AGG GGA-3′
	R: 5′-TTG GAT GTG AGA GGC GAA GAA-3′
*CAMK2D*	F: 5′-CTC TTG TTT TGC TGT TGG GCT-3′
	R: 5′-TGC TGA GAC ATT TGA GTC CGA-3′
*GAPDH*	F: 5′-CCAGGTGGTCTCCTCTGA-3′
	R: 5′-GCTGTAGCCAAATCGTTGT-3′
miR-212-3p	F: 5′-CGCGAGATCAGAAGGTGATT-3′
	R: 5′-GTCGTATCCAGTGCAGGGTCCGAGGTATTCGCACTGGATACGACAGCCAC-3′

## Results

### Identification of DEGs

**Figure 2:**
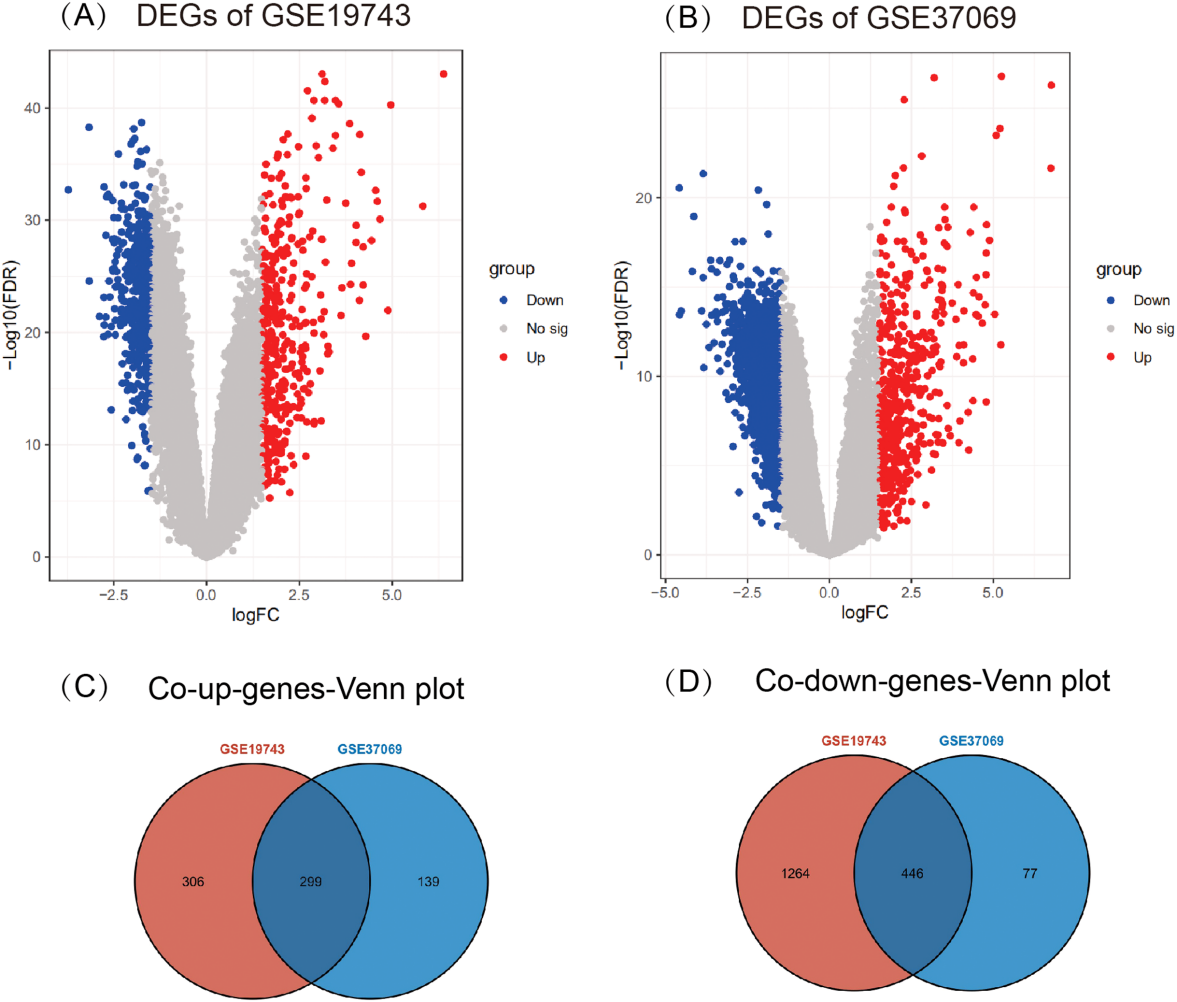
Results of different analyses in GSE19743 and GSE37069. Up-regulated DEGs are red while down-regulated DEGs are blue (|logFC| ≥ 1.5, *p* < 0.05). (A) GSE19743 had a total of 2,315 DEGs (605 up- and 1,710 down-regulated). (B) GSE37069 had a total of 961 DEGs (438 up- and 523 down-regulated). (C) Two hundred ninety-nine common up DEGs. (D) Four hundred forty-six common down DEGs.

A total of 2,315 DEGs (605 up- and 1,710 down-regulated) in GSE19743 (Fig. 2A) and 961 DEGs (438 up- and 523 down-regulated) in GSE37069 (Fig. 2B) were screened out. In the two gene sets, 745 DEGs were common expressed, 299 of which were common up genes (Fig. 2C) and 446 were common down genes (Fig. 2D).

### Immune infiltration analysis

**Figure 3:**
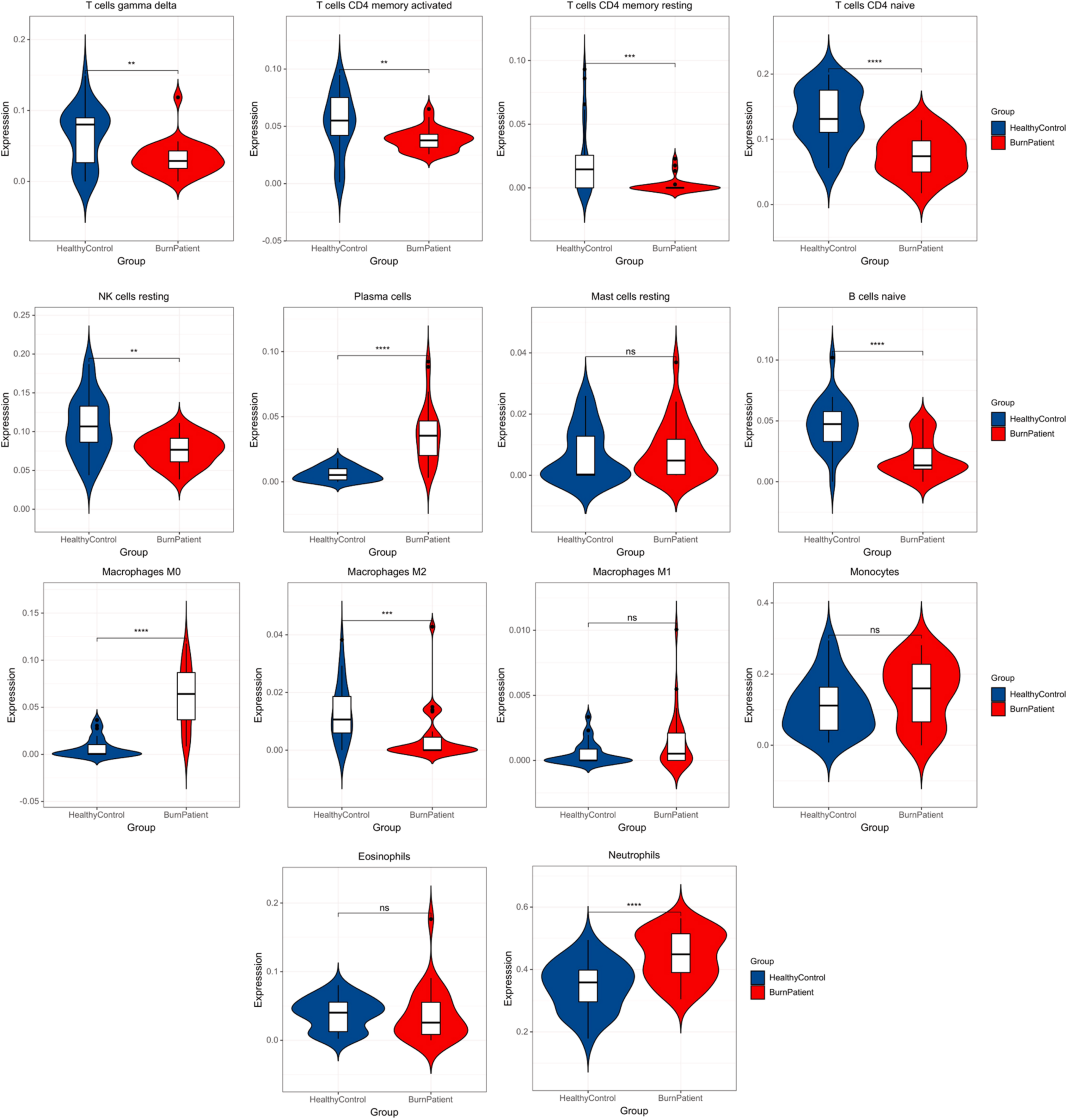
Results of immune infiltration analysis. Compared with the control group, burn patients had significantly decreased Th-cells (CD4+) (naive, memory resting, and memory-activated), T cells gamma delta, M2, B cells naïve, and NK cells resting, significantly higher M0 macrophages, plasma cells and neutrophils, and no difference in resting mast cells, M1, monocytes and eosinophils.

In the blood of burn patients, CD4^+^ cells (Th) (naive, memory resting, and memory-activated), T cells gamma delta, M2, B (naive), and NK (resting) cells decreased, while M0 macrophages, plasma cells, and neutrophils increased significantly (*p* < 0.05). Eosinophils, monocytes, M1 and resting mast cells showed no significance (*p* > 0.05) (Fig. 3).

### GO, KEGG pathway enrichment analysis, and GSEA of DEGs

#### GO enrichment analysis

**Figure 4:**
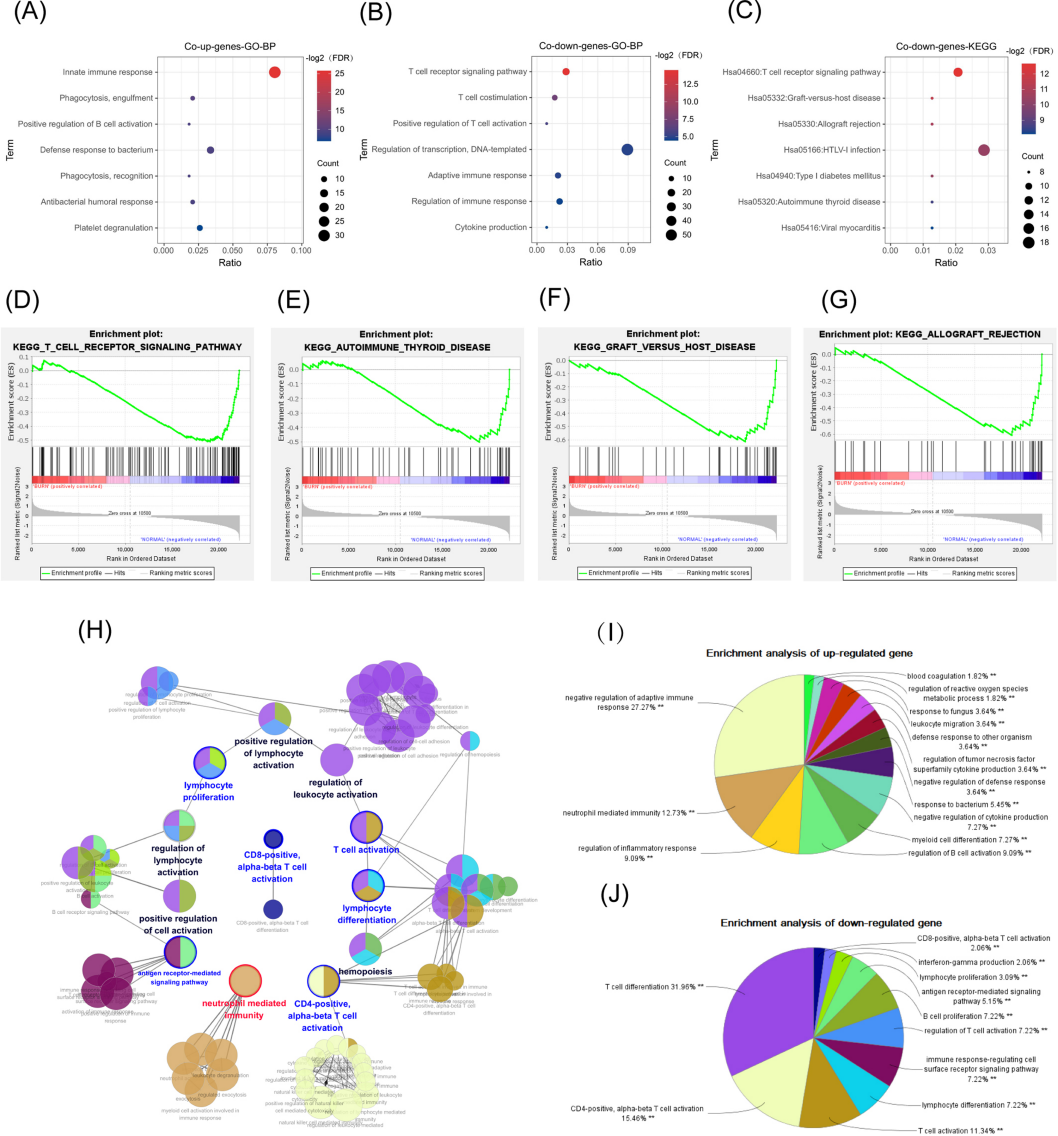
GO, GSEA, and KEGG results. The abscissa is the ratio of the number of genes in the pathway to the total genes. The redder the color, the smaller the FDR, and the larger the circle, the more enriched the genes. (A, B) Biological process in GO enrichment analysis showed that common up genes were mainly enriched in the innate immune responses such as neutrophil activation, while common down genes were mainly enriched in adapted immune responses such as the T-cell receptor signaling pathway. (C) The T cell receptor signaling pathway was also a result from the KEGG enrichment analysis in common down genes. (D) T-cell receptor signaling pathway. (E) Graft *versus* host disease. (F) Autoimmune thyroid disease. (G) Allograft rejection. (H, I) The network diagram of the interactions between the DEGs in the biological process enrichment analysis using the ClueGo plug-in of Cytoscape. (H) Each circle represents a pathway, and circles with similar functions form a module. The multiple colors in the same circle represent that this pathway participated in multiple modules. Module names are either red (meaning that the modules were mainly enriched with common up genes), blue (the modules were mainly enriched with common down genes), or black (the genes enriched in the modules did not have significant differences between the two groups). Genes in pathways related to the activation of T cells, especially Th-cells, were downregulated, while genes in pathways related to the innate immune response, such as neutrophil activation, were up-regulated. (I) The pie chart represents the proportion of genes in each pathway from the total number of Cluster 1 (common up genes) and (J) Cluster 2 (common down genes) genes.

According to our DAVID online analysis, the biological process in GO enrichment analysis showed that in the common up genes, there was a total of 11 enrichment terms (FDR < 0.05) that were mainly related to neutrophil activation. We visualized seven of them below (Fig 4A). However, in the common down genes, there were a total of seven enrichment terms (FDR < 0.05) that were mainly related to the proliferation and differentiation of T lymphocytes (Fig. 4B).

#### KEGG pathway analysis

There were 15 pathways, including the T-cell receptor (TCR) signaling pathway (FDR < 0.05), in the common down DEGs. The bubble chart shows the top seven statistically significant results (Fig. 4C) (FDR < 0.05).

#### GSEA

The GSEA analysis results showed that the GSE19743 gene matrix was mainly enriched in immune-related pathways (Figs. 4D–4G).

We successfully repeated the results of enrichment analysis using the ClueGO plug-in of CytoScape (Fig. 4H), confirming that the genes in Cluster 1 (common up DEGs) (Fig. 4I) were primarily related to the response to bacterial infection and neutrophil activation while genes in Cluster 2 (common down DEGs) (Fig. 4J) were primarily related to adaptive immune pathways, particularly T-cell activation pathways.

### Constructing IGR sets

We selected 168 IRGs from the GO, KEGG, and GSEA immune-related pathways for further analysis (Table 6).

**Table 6:** Immune-related genes in GO, KEGG, and GESA enrichment analysis.

ID	Term	FDR	Genes
GO:0045087	innate immune response	1.72E−06	*IGHM, CRISP3, IGHV3-23, GATA3, LY9, CLU, MALT1, LILRA5, IGKC, KLRC4-KLRK1, CLEC5A, S100A12, IGLC1, FYN, DEFA1B, PGLYRP1, JAK3, IGHA2, CAMP, AKIRIN2, FCER1G, CR1, KLRC2, DEFA4, SH2D1A, BMX, SRPK1, IGLL5, AIM2, VNN1, SLPI, LCK, LCN2, IGHD, LOC101060521, TLR7, PTX3, KLRD1, PADI4, TLR5, S100A8, NAIP, APOBEC3B*
GO:0006911	phagocytosis, engulfment	1.09E−05	*IGHM, IGLL5, FCER1G, IGKC, IGHV3-23, IGHD, IGLC1, IGHA2*
GO:0050871	positive regulation of B cell activation	2.69E−05	*IGHM, IGLL5, IGKC, IGHV3-23, IGHD, IGLC1, IGHA2*
GO:0042742	defense response to bacterium	3.54E−05	*FCER1G, ANXA3, IGHV3-23, HP, MPO, IGLL5, IGKC, IGHD, S100A12, IGLC1, TLR5, ELANE, CAMP*
GO:0006910	phagocytosis, recognition	4.29E−05	*IGHM, IGLL5, IGKC, IGHV3-23, IGHD, IGLC1, IGHA2*
GO:0006955	immune response	4.30E−05	*PTGER4, IL1RN, CXCL8, TCF7, IGHV3-23, LY75, CST7, LTB4R, FCAR, HLA-DMB, IGKC, ATP6V0A2, IGLC1, CTSG, DEFA1B, PGLYRP1, IGHA2, HLA-DPA1, CD96, IL1R2, GZMA, OSM, SERPINB9, GZMH, TGFBR3, CHIT1, AIM2, SLPI, CD8A, GPR183, IGHD, HLA-DPB1, HLA-DRA, CEACAM8, IL7R, IL18R1, HLA-DQB1*
GO:0050853	B cell receptor signaling pathway	4.38E−05	*IGHM, IGLL5, MEF2C, IGKC, PLEKHA1, LCK, IGHV3-23, BCL2, IGHD, NFATC2, IGLC1, IGHA2*
GO:0019731	antibacterial humoral response	5.51E−05	*IGHM, SLPI, DEFA4, RNASE3, DEFA1B, IGHA2, CAMP, LTF*
GO:0031295	T cell costimulation	7.04E−05	*LCK, TRAC, KLRC4-KLRK1, HLA-DPB1, CD28, HLA-DRA, CD3G, FYN, CD3D, HLA-DPA1, HLA-DQB1*
GO:0050870	positive regulation of T cell activation	3.20E−04	*LCK, HLA-DPB1, PRKCQ, CD47, MALT1, HLA-DPA1*
GO:0050852	T cell receptor signaling pathway	4.33E−04	*BTN3A1, TRAT1, TXK, TRAC, CD3G, GATA3, CD3D, MALT1, PSMA5, LCK, PSME4, HLA-DPB1, CD28, HLA-DRA, FYN, PRKCQ, HLA-DPA1, HLA-DQB1*
GO:0050900	leukocyte migration	5.50E−04	*FCER1G, ITGA4, ITGB3, MMP9, CD2, SLC7A6, CEACAM1, CEACAM6, LCK, OLR1, SIRPA, FYN, CEACAM8, CD47, CD44, CD177*
hsa05320	Autoimmune thyroid disease	8.48E−04	*HLA-DMB, HLA-DPB1, CD28, PRF1, HLA-DRA, GZMB, HLA-DPA1, HLA-DQB1*,
GO:0006355	regulation of transcription, DNA-templated	8.75E−04	*ZNF573, ZNF571, HP1BP3, RORA, AFF3, CCAR1, NR3C2, RPS6KA5, SCML1, LBH, ZNF805, ZNF83, ZNF529, ZXDB, ZNF207, ZNF567, ZNF600, PDE8A, MYBL1, ZNF320, MEF2C, RALGAPA1, ZFP3, ZNF160, SFMBT2, EBF1, RHOH, PAX5, ZNF92, EEF1D, RFX7, ZNF439, PRKCQ, ZNF677, ABCG1, ZNF432, ZNF550, SATB1, ZNF23, ZNF709, ZNF827, ZNF286B, STAT4, ZNF107, ZNF302, SCAI, ZNF420, ZFHX3, MTERF3, ZNF260, ZBTB10, NFATC2, NR1D2, ZNF736, ESF1, TARDBP*,
GO:0002250	adaptive immune response	9.56E−04	*EOMES, ERAP2, BTN3A1, TRAT1, TXK, SH2D1A, ERAP1, CRTAM, RNF125, CAMK4, GPR183, KLRC4-KLRK1, FYN*,
hsa04660	T cell receptor signaling pathway	1.31E−03	*NFATC2, CD3G, MAPK14, RASGRP1, CD3D, MALT1, DLG1, PPP3CC, LCK, CD8A, AKT3, CD28, FYN, PRKCQ*
GO:0050776	regulation of immune response	1.43E−03	*CD96, KLRB1, ITGA4, CD160, SH2D1A, TRAC, CRTAM, CD3G, CD3D, CD8A, KLRC4-KLRK1, KLRF1, KLRD1, CLEC2D*,
hsa05166	HTLV-I infection	1.44E−03	*STAT5B, IL1R2, NFATC2, ADCY3, RRAS2, CD3G, CD3D, ETS2, ELK4, DLG1, HLA-DMB, PPP3CC, LCK, IL2RB, AKT3, HLA-DPB1, TSPO, HLA-DRA, ANAPC5, JAK3, PRKACB, ATR, HLA-DPA1, HLA-DQB1*
hsa04612	Antigen processing and presentation	1.56E−03	*HLA-DMB, KLRC2, CD8A, KLRC3, HLA-DPB1, HLA-DRA, KLRD1, HLA-DPA1, HLA-DQB1*,
GO:0001816	cytokine production	1.75E−03	*MAF, TXK, NFATC2IP, NFATC2, RASGRP1, S100A8*,
GO:0006958	complement activation, classical pathway	1.81E−03	*IGHM, IGLL5, CR1, IGKC, IGHV3-23, IGHD, IGLC1, IGHA2, CLU*
hsa05332	Graft-*vs*-host disease	2.36E−03	*HLA-DMB, HLA-DPB1, CD28, PRF1, HLA-DRA, GZMB, HLA-DPA1, HLA-DQB1*
hsa04672	Intestinal immune network for IgA production	3.72E−03	*HLA-DMB, ITGA4, HLA-DPB1, CD28, HLA-DRA, HLA-DPA1, HLA-DQB1*,
hsa05330	Allograft rejection	4.97E−03	*HLA-DMB, HLA-DPB1, CD28, PRF1, HLA-DRA, GZMB, HLA-DPA1, HLA-DQB1*
hsa05321	Inflammatory bowel disease (IBD)	8.00E−03	*HLA-DMB, MAF, STAT4, HLA-DPB1, HLA-DRA, RORA, TLR5, IL18R1, HLA-DPA1, HLA-DQB1*
hsa04940	Type I diabetes mellitus	0.001100442	*HLA-DMB, HLA-DPB1, CD28, PRF1, HLA-DRA, GZMB, HLA-DPA1, HLA-DQB1*
hsa05152	Tuberculosis	0.001517592	*CAMK2D, CR1, FCER1G, MAPK14, MALT1, HLA-DMB, PPP3CC, LAMP1, AKT3, HLA-DPB1, BCL2, ATP6V0A2, HLA-DRA, CTSD, CAMP, HLA-DPA1, HLA-DQB1*
hsa05416	Viral myocarditis	0.00157646	*HLA-DMB, RAC2, HLA-DPB1, CD28, PRF1, HLA-DRA, FYN, HLA-DPA1, HLA-DQB1*
hsa05323	Rheumatoid arthritis	0.002170232	*HLA-DMB, CXCL8, ATP6V0A2, HLA-DPB1, CD28, HLA-DRA, HLA-DPA1, HLA-DQB1*,
hsa04650	Natural killer cell mediated cytotoxicity	0.003601034	*PPP3CC, LCK, SH2D1A, KLRC4-KLRK1, PRF1, NFATC2, GZMB, KLRD1, FYN*,

### Predicting miRNA-targeted genes and constructing miRNA–mRNA and common expression networks between key genes and key cycle immune cells

**Figure 5:**
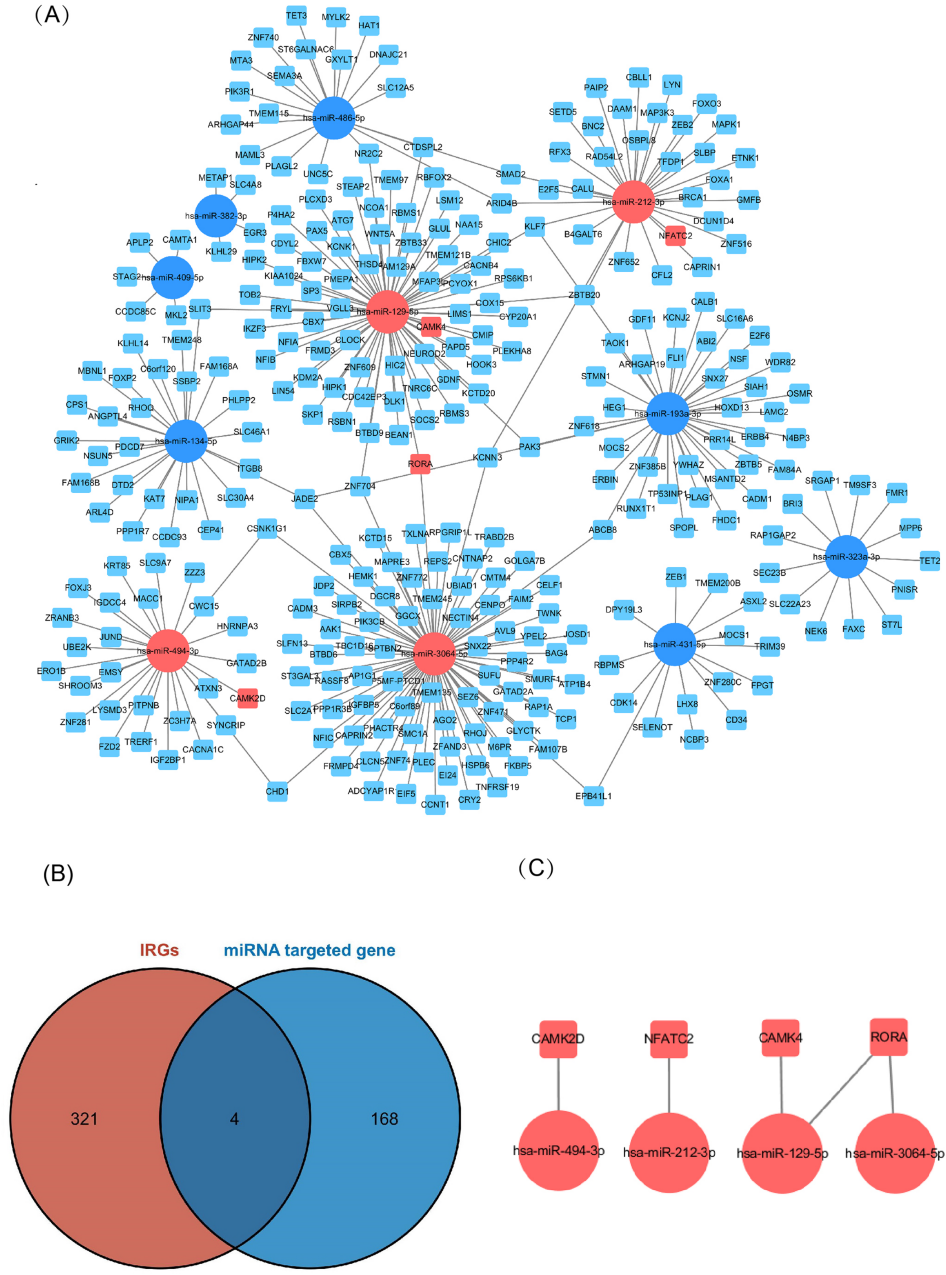
Network of miRNA and mRNA. (A) A total of 11 miRNAs were predicted using three databases at the same time, which are represented by circles in the network. The 11 differently expressed miRNAs had a total of 321 targeted mRNAs, which are represented by squares in the network. The red modules in the network were also immune-related genes, and the blue modules were not included in the immune-related genes. (B) There are 168 immune-related genes and 321 miRNA target genes. (C) There are four intersections among them.

**Figure 6:**
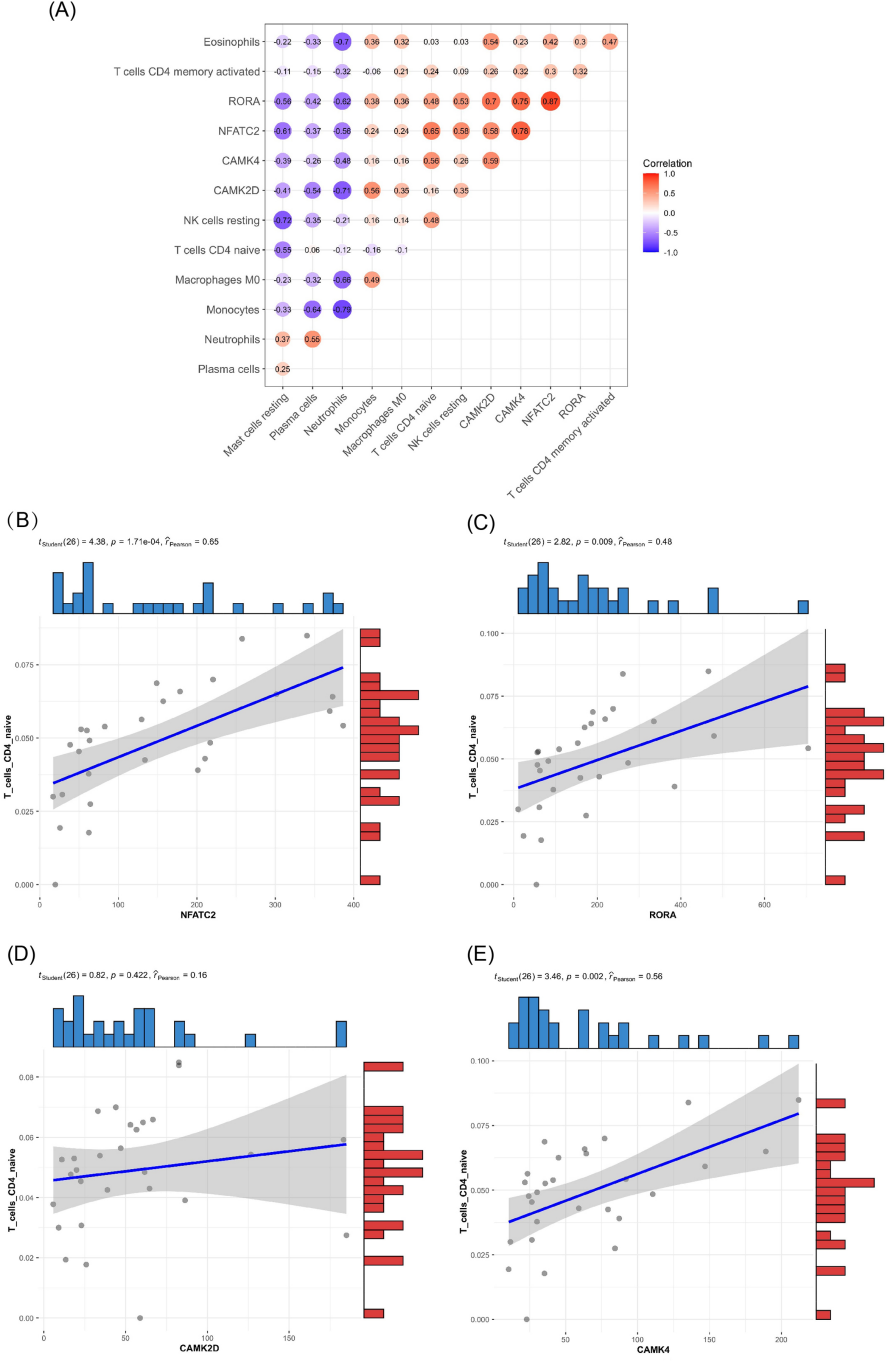
Relationship between key gene and CIC expression. (A) Common expression relationship between key genes and CICs. (B) Correlation between NFATC2 and CD4-naive T cells. (C) Correlation between RORA and CD4-naive T cells. (D) Correlation between CAMK4 and CD4-naive T cells. (E) Correlation between CAMK2D and CD4-naive T cells.

Of the DEMs we selected from thermal-stimulated epidermal stem cells, 34 were up-regulated and 13 were down-regulated (Table 7) (Rong & Liu, 2020) and there was a predicted total of 321 miRNA target genes. When looking at the intersection between IRGs and miRNA target genes, we identified four immune-related genes (key genes) (Figs. 5A and 5B) that may be regulated by miRNAs: *NFATC2*, *RORA*, *CAMK4*, and *CAMK2D* (Fig. 5C). Additionally, *NFATC2*, *RORA*, and *CAMK4* expression were related to the proportion of CD4-naive T cells (Fig. 6A) in burn patients. The expression profiles of key genes *NFATC2, RORA*, and *CAMK4* were correlated with CD4-naive T cells (Figs. 6B–6D).

**Table 7:** DEMs between thermal-stimulated epidermal stem cells and controls.

miRNA ID	log2 (fold change)	Adj. *p*. val
Up-regulated miRNA		
hsa-miR-4485-3p	4.2279	1.2 × 10^−7^
hsa-miR-1973	6.9388	2.1 × 10^−4^
hsa-miR-548j-5p	6.6339	7.2 × 10^−4^
hsa-miR-212-3p	6.6339	7.2 × 10^−4^
hsa-miR-4461	6.6339	7.2 × 10^−4^
hsa-miR-4510	6.5464	1.0 × 10^−3^
hsa-miR-3128	6.5464	1.0 × 10^−3^
hsa-miR-549a	6.4534	1.4 × 10^−3^
hsa-miR-494-3p	6.3539	1.9 × 10^−3^
hsa-miR-7641	6.3539	1.9 × 10^−3^
hsa-miR-1976	6.3539	1.9 × 10^−3^
hsa-miR-6868-3p	6.3539	1.9 × 10^−3^
hsa-miR-548u	6.3539	1.9 × 10^−3^
hsa-miR-2116-3p	6.2470	2.8 × 10^−3^
hsa-miR-3614-5p	6.2470	2.8 × 10^−3^
hsa-miR-744-3p	6.2470	2.8 × 10^−3^
hsa-miR-1287-5p	6.2470	2.8 × 10^−3^
hsa-miR-3064-5p	6.2470	2.8 × 10^−3^
hsa-miR-181b-2-3p	6.2470	2.8 × 10^−3^
hsa-miR-3176	6.1313	4.1 × 10^−3^
hsa-miR-516b-5p	6.0058	6.1 × 10^−3^
hsa-miR-338-5p	6.0058	6.1 × 10^−3^
hsa-miR-4746-5p	6.0058	6.1 × 10^−3^
hsa-miR-20a-3p	6.0058	6.1 × 10^−3^
hsa-miR-3688-3p	6.0058	6.1 × 10^−3^
hsa-miR-3179	6.0058	6.1 × 10^−3^
hsa-miR-6514-3p	5.8684	9.2 × 10^−3^
hsa-miR-1237-3p	5.8684	9.2 × 10^−3^
hsa-miR-431-5p	5.8684	9.2 × 10^−3^
hsa-miR-382-3p	5.8684	9.2 × 10^−3^
hsa-miR-134-5p	5.8684	9.2 × 10^−3^
hsa-miR-4659a-3p	5.8684	9.2 × 10^−3^
hsa-miR-409-5p	5.8684	9.2 × 10^−3^
Down-regulated miRNA		
hsa-miR-4520-5p	−6.8054	2.9 × 10^−4^
hsa-miR-4661-5p	−6.4835	1.0 × 10^−3^
hsa-miR-191-3p	−6.3904	1.4 × 10^−3^
hsa-miR-129-5p	−6.3904	1.4 × 10^−3^
hsa-miR-147b	−6.2908	1.9 × 10^−3^
hsa-miR-6868-3p	−6.2908	1.9 × 10^−3^
hsa-miR-323a-3p	−6.1839	2.8 × 10^−3^
hsa-miR-6515-5p	−6.1839	2.8 × 10^−3^
hsa-miR-1295a	−6.1839	2.8 × 10^−3^
hsa-miR-1248	−6.1839	2.8 × 10^−3^
hsa-miR-193a-3p	−6.0685	4.1 × 10^−3^
hsa-miR-1294	−6.0685	4.1 × 10^−3^
hsa-miR-149-3p	−6.0685	4.1 × 10^−3^
hsa-miR-6887-3p	−5.9430	6.1 × 10^−3^
hsa-miR-510-5p	−5.9430	6.1 × 10^−3^
hsa-miR-486-5p	−1.7053	7.4 × 10^−3^
hsa-miR-2277-5p	−5.8053	9.2 × 10^−3^
hsa-miR-6806-3p	−5.8053	9.2 × 10^−3^
hsa-miR-4683	−5.8053	9.2 × 10^−3^
hsa-miR-4504	−5.8053	9.2 × 10^−3^
hsa-miR-29b-2-5p	−5.8053	9.2 × 10^−3^

### Validating key genes in different gene sets

**Figure 7:**
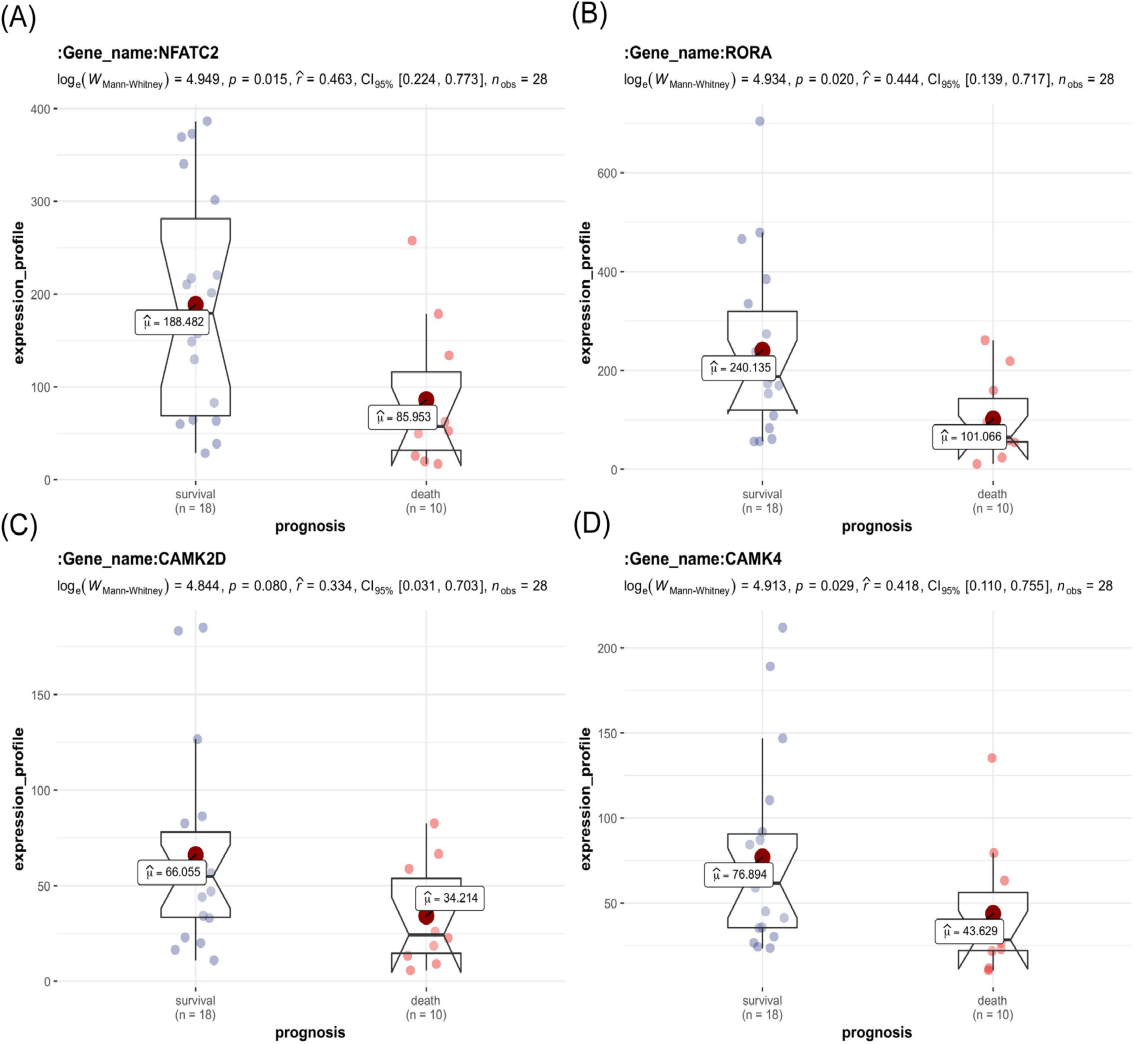
Differences in the expression profiles of key genes between surviving and non-surviving patients.

**Figure 8:**
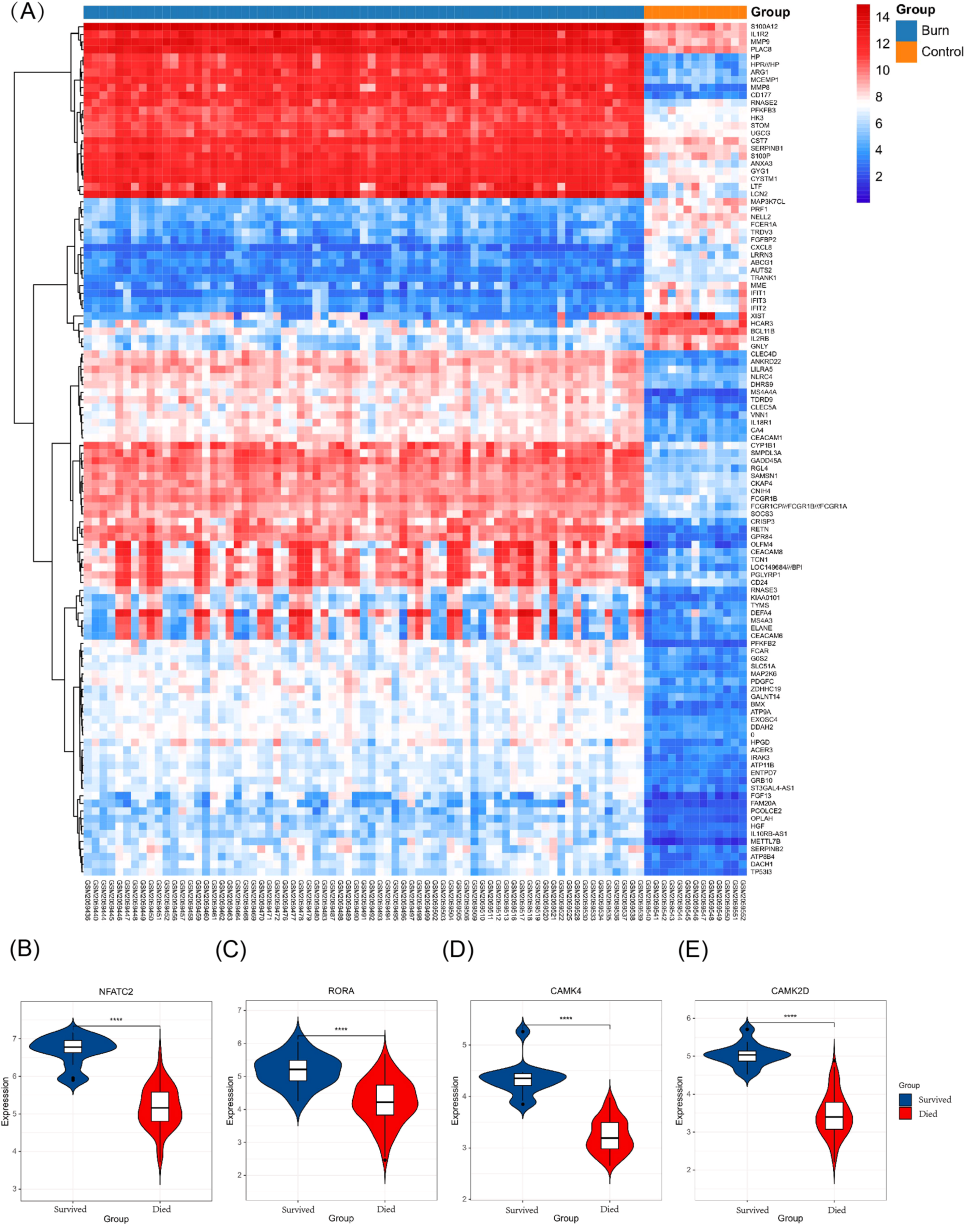
Different expression profiles between burn patients and health controls in GSE77791. (A) Heat map of GSE77791 (Top 100). (B–E) Key genes were significantly different between survival and non-survival patients.

**Figure 9:**
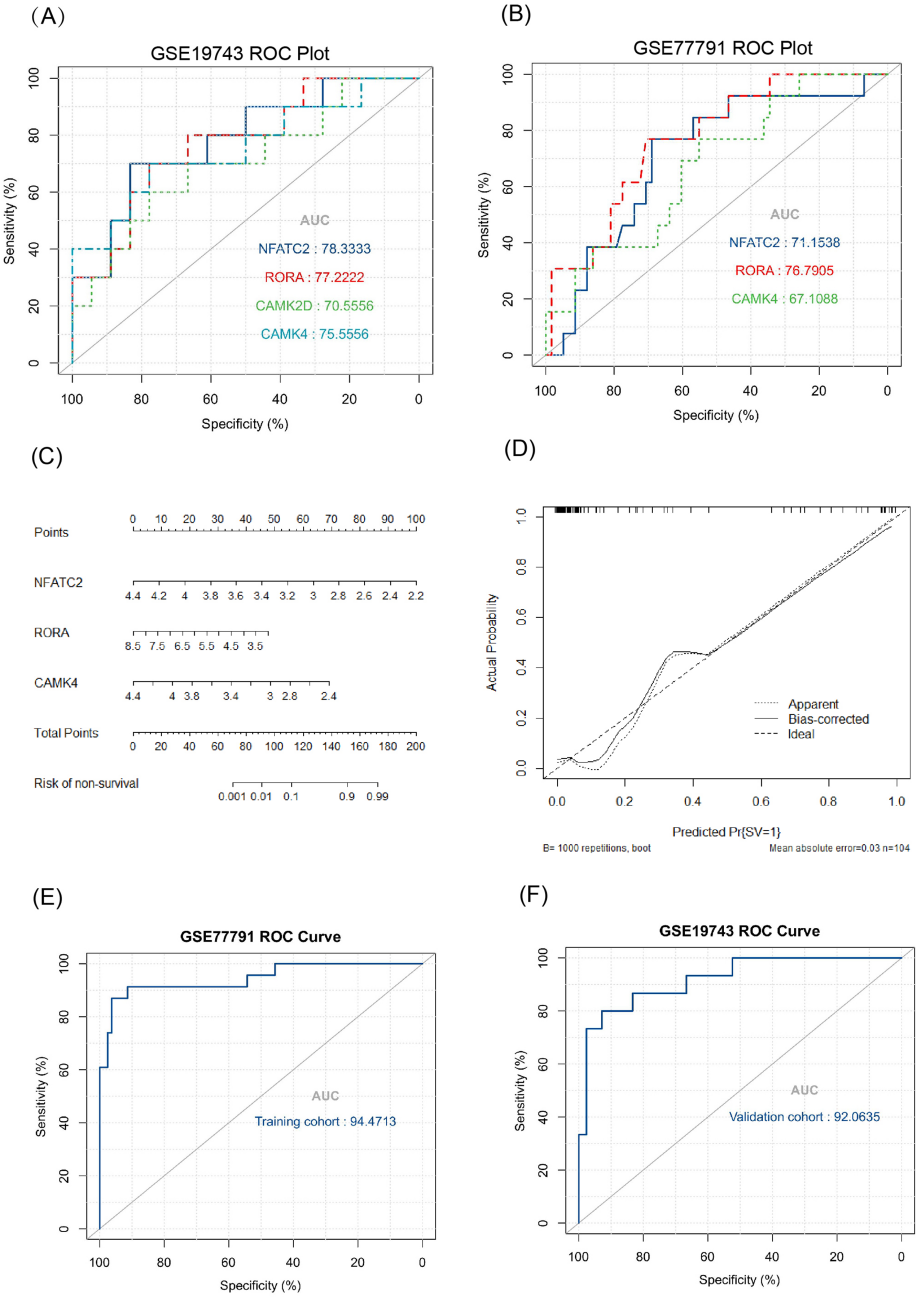
Results of the regression model. (A, B) ROC curve of key genes in burn patients in GSE19743 and GSE77791. (C) The nomogram of the multivariate logistic regression, (D) calibration, and (E) ROC curves in GSE77791. (F) Validation results of logistic regression model in GSE19743 (*p* < 0.05).

The key genes showed a significant difference in survival and non-survival patients in GSE19743 (Fig. 7). In GSE77791, the gene profile expression was significantly different between burn patients and healthy controls (Fig. 8A), and the expression of *NFATC2, RORA*, *CAMK4*, and *CAMK2D* also differed significantly between patients who survived and those who died (*p* < 0.05) (Figs. 8B–8E). In GSE19743, the area under the curve (AUC) values of the ROC curves in *NFATC2, RORA, CAMK4*, and *CAMK2D* were 0.783, 0.772, 0.755, and 0.705, respectively (*p* < 0.05), indicating a better predictor of prognosis (Fig. 9A). In GSE77791, the AUC values of *NFATC2, RORA*, and *CAMK4* were 0.712, 0.768, and 0.671, respectively (*p* < 0.05), which also suggests a better prognostic predictor (Fig. 9B).

### Constructing a survival related risk regression model

We constructed a logistic regression model using 104 burn samples (81 survival and 23 non-survival), as well as a nomogram (Fig. 9C) and calibration curves (Fig. 9D). The AUC value of the ROC curves of the regression model was 0.945 (*p* < 0.05) (Fig. 9E). We measured the model’s prognostic prediction ability in the external cohort (GSE19743) and the AUC value indicated excellent prediction abilities (AUC: 0.920) (*p* < 0.05) (Fig. 9F).

### Expression level of key genes and their miRNA

**Figure 10:**
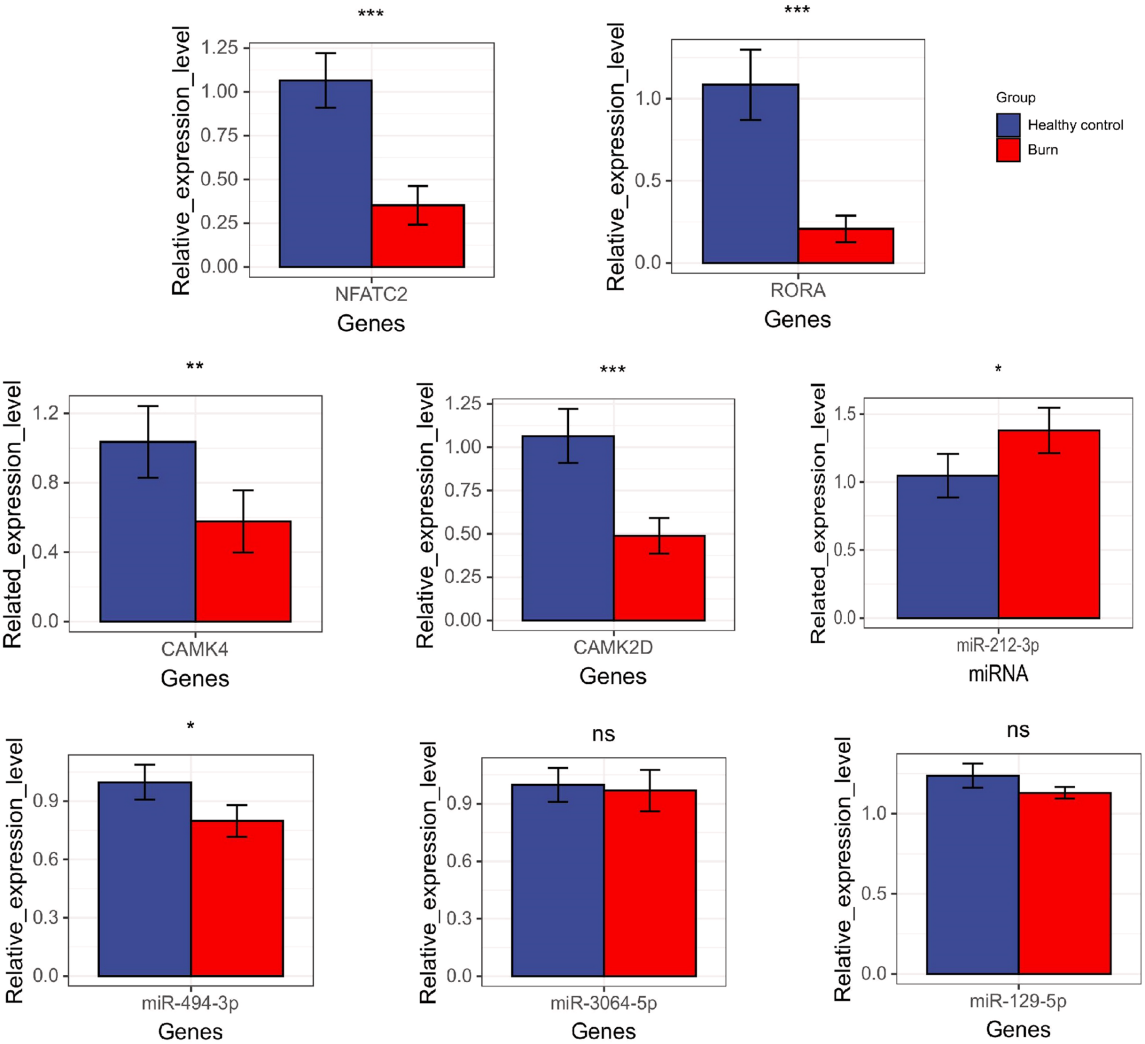
The results of qRT-PCR regarding NFATC2, RORA, CAMK4, CAMK2D, mir-212-3p, miR-3064-5p, miR-494-3p, and miR-129-5p. “*” means (*p* < 0.05), “**” means (*p* < 0.01), “***” means (*p* < 0.001), and “ns” means no difference.

The results showed that the expression of *NFATC2, CAMK4, RORA*, *CAMK2D*, and miR-494-3p decreased in burn patients’ blood (*p* < 0.05). However, miR-212-3p expression increased (*p* < 0.05) and t miR-3064-5p and miR-129-5p expression showed no significant differences between burn and normal patients (p > 0.05). *NFATC2, CAMK4, RORA*, and miR-212-3p expression was consistent with data from RNA-seq (Fig. 10).

## Discussion

Although previous studies explored the changes in a specific subtype of immune cells after burns, none of them, to our knowledge, used IRGs to predict prognosis. Severe burns are further complicated by a reduced numbers of T cells and dysregulated differentiations of Th-cells in the blood and spleen (O’Sullivan et al., 1995; Tschöp et al., 2009; Zhang et al., 2017; Deveci et al., 2000). In this study, we found that the number of neutrophils, plasma, and M0 macrophages increased, but T cells gamma delta, M2, B cells naive, NK cells resting and Th (CD4^+^) (naive, memory resting, and memory activated) cells, which are effector cells of the adaptive immune response, significantly decreased in burn patients. This persisted for a considerable period of time after the burn injury (Tschöp et al., 2009). Previous studies also showed that this immune-cell disorder, characterized by an overpowering inflammatory response and a state of adaptive immunosuppression, was an important factor for compromised immunity following burn injury (Samonte et al., 2004). Thrombopenia, burn-severity models, and immune cells have been used to predict prognosis, but they are insufficient. We determined that the IRGs NFATC2, RORA, and CAMK4 were significantly associated with prognosis, and performed better than white blood cells, platelets, cytokines, burn areas, and score scales. Immunotherapy can improve the function of immune cells and reduce deaths caused by infection, but available targets are still few (Toliver-Kinsky et al., 2003; Williams et al., 2009). In our research, we found that NFATC2, RORA, and CAMK4 were co-expressed with Th cells in burn patients. Th-cells are associated with important the adaptive immune response, which is mainly involved in adaptive immune disorders after burns (Patenaude et al., 2005). This also means that these genes are potential targets for immunotherapy and a meaningful direction for future research.

In GO terms, common up DEGs were mainly enriched in innate immune response, which is characterized by the activation of neutrophils, while common down DEGs were in the TCR signaling pathway that controls the development of T cells. In KEGG analysis, common down DEGs were mainly enriched in the TCR signaling pathway (FDR < 0.05) and other T-cell activation-related pathways. Additionally, we repeated the results of enrichment analysis using ClueGo. Biological process enrichment analysis *via* ClueGo showed that the common up DEGs were mainly enriched in the neutrophil-mediated inflammatory response and antimicrobial response pathways. The common down DEGs were mainly enriched in pathways related to lymphocyte (particularly T-cell) activation and proliferation, such as the TCR signaling pathway, lymphocyte activation, and antigen receptor presentation. These results are consistent with previous experiments that showed an excessive inflammatory response and suppression of the adaptive immune response in humans and mice following burn injury (Zhang et al., 2017). However, in this study, we explored the possible mechanism from the level of the pathway and molecular mechanisms.

Our immune infiltration analysis determined that in burn patients, the changes in the proportion of 14 types of circulating immune cells, especially Th cells, neutrophils, and macrophages, were probably relevant to immune dysfunction. Th is a key adaptive immune cell according to characteristic cytokines and lineage transcription factors, Th-cells (CD4^+^) are classified as Th1 (IFN-γ and T-bet), Th2 (IL-4/IL-5/IL-13 and GATA3), Th17 (IL-17/IL-22 and RORγt), or Treg (IL-10/TGF-β/IL-35 and Foxp3). Th1 secretes IFN-γ and IL12, which mainly plays a pro-inflammatory role. Th2 secretes IL-4, IL-5, and IL-13 (Gurram & Zhu, 2019), which can play an anti-inflammatory role. Th17 secretes IL-17A, which plays a crucial role in the mucosal antibacterial response (Annunziato, Romagnani & Romagnani, 2015; Park et al., 2005). Treg secretes IL-10, TGFβ, and IL-35, which can suppress other immune cells (Shevach, 2009) and inhibit the antigen-presenting function of dendritic cells (DCs) (Shevach & Thornton, 2014). Th-cell proliferation and differentiation disorders, such as suppressed Th1 differentiation, increased Th2 differentiation, and an augmented number of Th17 and Treg cells, are thought to play an important role in adaptive immunosuppression following burn injury (O’Sullivan et al., 1995; Valvis et al., 2015; MacConmara et al., 2011; Hunt et al., 1998; Rendon & Choudhry, 2012). Furthermore, burns induce the apoptosis of T cells, leading to a decrease in the number of T cells (Zhang et al., 2018). Finally, burns lead to the impaired secretion of Th cytokines, for example, a decrease in pro-inflammatory cytokines IFN-γ, IL-2, and IL-12, which can induce the differentiation of Th1 cells. Meanwhile, anti-inflammatory cytokines IL-4, IL-10, and IL-1 increased. IL-4 can induce Th2 which produces IL-10, through which Th2 cells can inhibit antigen presentation *via* APCs and induce reduction of inflammation (O’Sullivan et al., 1995; Beckmann et al., 2020). These cytokine alterations eventually lead to immune dysfunction characterized by Th1/Th2 and Th17/Treg alterations (Zhang et al., 2018). Neutrophils and macrophages are also important effector cells in the inflammatory response. In burn patients, neutrophils and macrophages are activated and mediate the inflammatory response to clear necrotic tissue and exert anti-infective effects (Schwacha, 2003; von Müller et al., 2020). However, the adaptive immune response is diminished in burn patients, and it is known that neutrophils and immature neutrophils in tumors can suppress Th-cell activity and recruit Treg cells to promote immunosuppression, which may be the same mechanism in burn patients (Kinoshita et al., 2011; Aarts et al., 2019; Hampson et al., 2017). At the same time, excessive macrophages can inhibit the function of Th-cells in burn patients (Schwacha, 2003; Luo et al., 2005). We speculated that the overactivation of inflammatory cells in burn patients may lead to the imbalance of immune cells, which may be an important factor of post-burn immunosuppression. However, recent studies on these immune cell abnormalities have mainly focused on cells and cytokines, and there are few studies at the gene level, which are the main causes of pathological changes. The results of this analysis were consistent with previous experiments at the cellular level, but we revealed more pathways and molecular abnormalities at the gene level, which will be helpful for future experimental studies.

*NFATC2, RORA, CAMK4*, and *CAMK2D* were immune-related DEGs and also target genes of miRNAs that were differentially expressed in thermal-stimulated epidermal stem cells. We identified four mRNA-miRNA pairs: *NFATC2* and miR-212-3p, *RORA* and miR-3064-5p, *RORA* and miR-129-5p, and *CAMK2D* and miR-494-3p. Our correlation analysis showed that *NFATC2*, *RORA*, and *CAMK4* were positively correlated with the proportion of naive CD4^+^ T cells, all of which were downregulated.

*NFATC2* is a member of the NFAT family, and a decrease of NFAT, caused by events such as the reduction of IL-2, can affect the production of cytokines in the Th-cells of burn patients (Choudhry et al., 2002). *NFATC2* is expressed in Th1, Th2, Th17, and Treg cells as a downstream signaling molecule of the TCR signaling pathway and interacts with multiple Th-cell developmental transcription factors such as AP1, FOXP3, and BATF to regulate cytokine production (Mehta et al., 2005; Wu et al., 2006). *NFATC2* increases the expression of IL17-A and IL21, thereby enhancing Th17 cell-mediated inflammatory response (Kaminuma et al., 2012; El‐Said et al., 2019). Defects in *NFATC2* lead to a decreased thymocyte count, impaired T-cell proliferation, and decrease of cytokines such as IL-4 and IL-17A that are characteristic of Th2 and Th17 in mice (Gomez-Rodriguez et al., 2009). However, when Treg cells and their characteristic cytokines, IL-10 and TGF-β, are increased, this enhances immunosuppression (Rathod et al., 2020). Additionally, *NFATC2* deficiency showed the same trend of change in IL-21 as miR-320c (El‐Said et al., 2019). In this study, *NFATC2* significantly decreased in burn patients and was common expressed with naive CD4^+^ T cells. However, to our knowledge, no one has yet conducted an in-depth study on *NFATC2* and its targeted miRNA, which may be a key gene or target of immunotherapy for Th-cell dysfunction in post-burn patients. Therefore, *NFATC2* merits further investigation with respect to immunosuppression in burn patients.

Both *RORA* and *RORγt* belong to the retinoid acid receptor-related orphan receptor family. *RORγt* is an important transcription factor that regulates Th17 differentiation. *RORA* can synergize with *RORγt* to promote differentiation and the function of Th17 cells. A combined lack of *RORγt* and *RORA* can completely eliminate IL-17 expression (Yang et al., 2008). IL-17 is a characteristic cytokine of Th17 cells that exerts mucosal immunity. Impaired immune barriers in the intestinal and respiratory mucosa played a key role in bacterial translocation after burn injury (Grimes et al., 2016). In this study, *RORA* was significantly downregulated in burn patients. *RORA* may be a key molecule or biomarker for this pathological process.

*CAMK4* and *CAMK2D* are both calcium/calmodulin-dependent kinases that are members of the *CAMK* family. This family is a downstream molecule of the second messenger, Ca^2+^, which plays an important role in the activation of T cells. *CAMK4* is highly expressed in CD4^+^ T cells (Hanissian et al., 1993). When the TCR signaling pathway is activated, the concentration of Ca^2+^ rises, activating *CAMK4* downstream, promoting the activation of downstream pathways, and amplifying downstream signals for T-cell activation (Hanissian et al., 1993; Hook & Means, 2001). *CAMK4* can negatively regulate IL-2 transcription to promote IL-17 transcription in Th17 cells. Mice lacking *CAMK4* inhibit IL-17 production through the Akt/mTOR/IL-17A axis, which impacts the development of Th17 cells (Anderson & Means, 2002; Koga et al., 2014; Koga & Kawakami, 2018). In addition, *CAMK4* activates the phosphorylation of CREB to regulate NFAT transcriptional activity (Yu, Shih & Lai, 2001; Sato et al., 2006) and regulates the activity of transcription factor ROR *via* the *CAMKK/CAMK4* cascade response (Kim & Leonard, 2007). In adults, silencing of the *CAMK4* gene reduces the mRNA levels of IL-17, IL-22, and *RORγ* and inhibits Th17 differentiation. *CAMK4* function-deficient mice showed enhanced Treg cell activity (Koga et al., 2014; Zhan et al., 2020; Koga et al., 2014; Koga et al., 2012), which disrupted the balance of Th17 and Treg cells, leading to a disturbed balance between mucosal immunity and immunosuppression. Apparently, *CAMK4* is essential to promote the function of Th17 cells and suppress Treg cells. In this study, *CAMK4* expression was found to be significantly downregulated, which may be an important mechanism and target of immunotherapy in burn patients with immunosuppression.

The AUC values of the ROC curves revealed that *NFATC2, RORA, CAMK4*, and *CAMK2D* showed a good predictive ability for patient survival status in GSE19743. However, in the logistic hazards model (GSE77791), only *NFATC2, RORA*, and *CAMK4* had a superb ability to predict prognosis (AUC:0.945). Therefore, *CAMK2D (p* = 0.781*)* was excluded from the ROC curves in GSE77791. Furthermore, there were significant differences in the expression levels of *NFATC2, RORA*, and *CAMK4* between the survival and non-survival groups (*p* < 0.05). Sex (*p* = 0.278), age (*p* = 0.270), and TBSA (*p* = 0.248) were excluded in the logistics hazards model, which means that *NFATC2* (*p* < 0.05, OR = 0.006), *RORA* (*p* < 0.05, OR = 0.427), and *CAMK4* (*p* < 0.05, OR = 0.004) are independent factors for predicting prognosis. A nomogram was established to predict the survival probability of burn patients in GSE19743, and ROC and calibration curves showed that the model performed outstandingly in predicting prognosis (AUC:0.920) in a validation cohort. The above studies convincingly showed that that *NFATC2*, *RORA*, and *CAMK4* are all important genes that were related with the development and proliferation of T cells and can be used as biomarkers to predict prognosis. However, the specific role of these genes in burn injury has not been thoroughly studied. Additionally, the above genes may be regulated by miR-212-3p (*NFATC2*), miR-3064-5p (*RORA*), miR-129-5p (*RORA, CAMK4*), and miR-494-3p (*CAMK2D*), and we found that the expression of *NFATC2*, *CAMK4* and *RORA* were down-regulated and miR-212-3p was up-regulated in burn patients. Although some miRNAs were not differentially expressed in burn patients, the interaction network between miRNA and mRNA was only a process of narrowing the gene range and the mRNAs were down regulated in 213 burn patients and verified by PCR. This does not affect the fact that mRNA is differentially expressed *in vivo* nor its ability as a prognostic biomarker. This finding will guide subsequent research that will explore immunotherapy targets. For example, some miRNAs may need only a small amount to play a role, or there are other regulatory networks to be further explored. In all, *NFATC2, RORA*, and *CAMK4* can predict prognosis and have the potential to restore immune function.

There have been previous bioinformatic studies on burns. In the GSE7404 gene set, the authors studied DEG changes after burns (Gao et al., 2016) and found that DEGs were related to immune dysfunction. However, this study was conducted on mice, hence it was difficult to apply these findings to human subjects. Others studied the sequencing data of burn patients in GSE77791 and also believed that the immune function of burn patients was suppressed (Fang et al., 2020). In addition, Li et al. (2016) studied the GSE19743 data and found DEGs were enriched in immune-related pathways. However, these two studies neither analyzed the interaction and activation status in the pathways nor revealed the correlation between key genes and prognosis, and the relationship between DEGs and cell phenotypic changes remains unknown. Some scholars have tried to build a prognostic model using a pattern of immune cells, such as red blood cells (RBCs), WBCs including neutrophils, monocytes, and lymphocytes, and platelets. However, they are only limited to a few types of cells, and sensitivity and specificity of the model did not perform very well (Osuka et al., 2019). There were also scholars who have studied the relationship between cytokines, Baux score, burn area, complications, and prognosis, but those predictors were not able to accurately predict the prognosis of burn patients and are highly subjective in practical application (Kraft et al., 2012; Prasad, Thode & Singer, 2020; Hur et al., 2015).

To our knowledge, ours is the first study in which three gene sets, including a total of 213 burn patients, were analyzed using bioinformatics. We described the overall pattern of immune cells in the blood of patients following burns. We found that the functional annotation of DEGs was related to dysfunction in the Th-cells and activated an inflammation response. We also identified the key genes that were associated with the decrease of Th-cells and performed well during the predicting prognosis. According to qRT-PCR, the expression of *NFATC2, CAMK4, RORA*, and miR-212-3p were consistent with the data from RNA-seq. Therefore, we hypothesized that these genes are likely to be prognostic biomarkers and are beneficial for subsequent research that will explore immunotherapy targets to improve the immune function of post-burn patients.

Our study had some limitations. The clinical data in this study was insufficient. We need more clinical data on shock *vs* non-shock, treatment methods, and survival times to give our model strength and credibility, and we need more prognostic markers to comprehensively assess the prognosis of burn patients. Further, the number of patients for the analysis of correlation between mRNA and clinical outcomes was not large enough and there was also a lack of clinical data to explore the relationship between miRNA expression and prognosis. More molecular biology, cell, and animal experiments are required to further confirm the interaction between mRNA and miRNA and the value of key genes as immunotherapy targets and prognostic biomarkers. We hope these results can improve prognosis, guide future studies with larger sample sizes and detailed clinical data to confirm the prognostic ability of key genes, and guide the next experiment that will explore immunotherapy biomarkers in post-burn immunosuppression.

## Conclusion

Using the bioinformatics approach, we identified the key cell, Th-cell, that likely functions in immunosuppression in burn patients. Based on the mRNA-miRNA network, we found key genes and constructed logistic hazard regression that performed well in predicting the survival in burn patients. In this study, we also found that the trends in the expression levels of NFATC2, RORA, and CAMK4 were the same as the proportion of Th-cells in the blood of burn patients and the lower expression of those genes were correlated with death in burn patients. Therefore, we hypothesized that these genes are potential immunotherapy targets and prognostic biomarkers in post-burn immunosuppression.

## Supplemental Information

10.7717/peerj.12680/supp-1 Supplemental Information 1 Code of R.

10.7717/peerj.12680/supp-2 Supplemental Information 2 Raw of qPCR.

10.7717/peerj.12680/supp-3 Supplemental Information 3 Raw data of qPCR.

## Abbreviations

DEGs: differentially expressed genes
GO: Gene Ontology
CICs: circulating immune cells
KEGG: Kyoto Encyclopedia of Genes and Genomes
IRGs: Immune-related genes
DEMs: differential expression of miRNAs
GSEA: Gene set enrichment analysis
TCR: T cell receptor
ROC: receiver operating characteristic

## Additional Information and Declarations

### Data Availability

The following information was supplied regarding data availability: The code is available in the Supplemental File.
