# Supplementary figures and images for: Identifying changes in immune cells and constructing prognostic models using immune-related genes in post-burn immunosuppression
Source: PeerJ. 2022 Jan 13;10:e12680. doi: 10.7717/peerj.12680 (PMC8761370; doi:10.7717/peerj.12680)

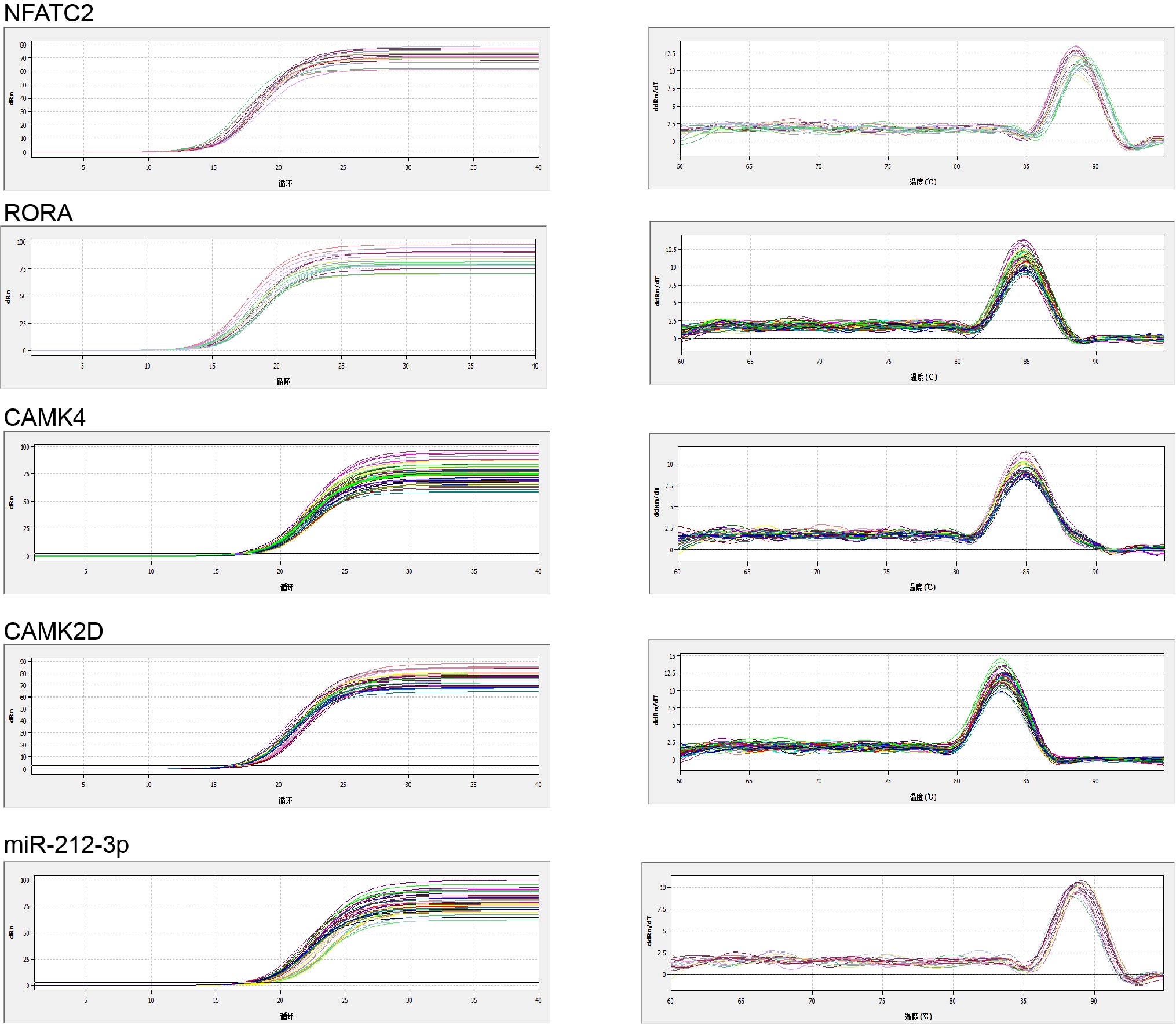

Supplement: Supplemental Information 2 [file peerj-10-12680-s002.jpg]
